# ﻿*Cladopusyangjiangensis* (Podostemaceae), a new species from Guangdong, South China, redefining the phylogenetic relationships within *Cladopus*

**DOI:** 10.3897/phytokeys.249.140342

**Published:** 2024-12-02

**Authors:** Miao Zhang, Xiu-Ting Liu, Min Tian, Zhang-Xue Chen, Ying-Lin Huang, Guo-Di Chen, Bing-Hua Chen

**Affiliations:** 1 College of Life Sciences, Fujian Normal University, Fuzhou 350117, China; 2 Ting Jie Natural History Studio, Guangzhou 510000, China; 3 The Public Service Platform for Industrialization Development Technology of Marine Biological Medicine and Products of the State Oceanic Administration, Fujian Key Laboratory of Special Marine Bioresource Sustainable Utilization, Southern Institute of Oceanography, College of Life Sciences, Fujian Normal University, Fuzhou 350117, China; 4 Yangjiang Municipal People's Government Taiwan, Hong Kong and Macao Affairs Bureau, Yangjiang 529500, China

**Keywords:** Biodiversity, chloroplast genome, morphology, phylogeny, Podostemaceae

## Abstract

This paper introduces *Cladopusyangjiangensis*, a newly identified species that enriches our understanding of the diversity of the Podostemaceae in East Asia. Distinctive in its morphological traits, this species is characterized by the region’s longest flowering shoots and exhibits a high number of elongated leaves per cluster, along with relatively slender roots. Phylogenetic analyses using Maximum Likelihood and Bayesian Inference methods on plastome and *matK* sequences confirm *C.yangjiangensis* as a distinct species. It forms a clade with *C.fukienensis*, its closest relative, together branching off from *C.austrosinensis*. The plastome of *C.yangjiangensis* is 132,818 bp in length, comprising two inverted repeat regions of 20,881 bp, which are separated by large and small single-copy regions of 78,713 and 12,343 bp, respectively. Genetic analysis reveals the extensive loss of the *ycf1* and *ycf2* genes in the chloroplast genome, a trait common to the Podostemaceae, suggesting adaptations to environmental conditions or gene transfers to nuclear or mitochondrial genomes. This study improves the clarity of phylogenetic relationships in previous studies and underscores the importance of continued taxonomic and phylogenetic research.

## ﻿Introduction

The Podostemaceae family is a distinctive group within the angiosperms, notable for its species richness among aquatic flowering plants. These plants primarily inhabit tropical and subtropical regions, with some species extending into temperate zones (Cook 1996; Kato 2016). Podostemaceae species are uniquely adapted to rocky habitats within waterfalls or rapids. During high water levels in summer, they grow vigorously in their vegetative form submerged in water. As water levels recede in autumn and winter, these plants emerge to flower, fruit, and complete their life cycle (Koi and Kato 2012; Kato et al. 2019). Despite the similarity in habitats worldwide, the morphology of Podostemaceae is notably unique and diverse (Koi and Kato 2012). A striking feature is their roots, which function primarily as adhesive organs, contrasting with the anchoring and absorptive roots of terrestrial plants. The stems, leaves, and flowers are also determined by the root system (Koi and Kato 2020).

The Podostemaceae family is composed of three subfamilies: Tristichoideae, Weddellinoideae, and Podostemoideae, encompassing approximately 51 genera and 350 species (Kita and Kato 2001; Koi et al. 2022). Tristichoideae and Weddellinoideae are relatively smaller groups, whereas Podostemoideae is the most species-rich and widely distributed subfamily (Costa et al. 2011; Katayama et al. 2022). Tristichoideae are characterized by their prominent perianth, while Podostemoideae are distinguished by their very small, scale-like or filamentous perianth and membranous spathe covering the bud (Kita and Kato 2001).

Within the Podostemoideae subfamily, the genus *Cladopus* is notable for its flat, nearly cylindrical or strap-like roots, digitate and rough-surfaced bracts that are 4–7-lobed, one or rarely two stamens, and smooth-surfaced capsules (Kato 2006, 2008; Koi and Kato 2012). Phylogenetically, *Cladopus* is closely related to *Paracladopus*, sharing a common ancestor with *Hanseniella*, *Hydrodiscus*, and *Thawatchaia* (Koi and Kato 2012; Koi et al. 2012). Geographically, *Cladopus* species are found in Southeast Asia, East Asia, Malaysia, and Australia (Kato et al. 2019). In China, *Cladopus* species have been reported in Fujian, Guangdong, Hainan, Guangxi, and Hong Kong (Kato et al. 2017; Li et al. 2024).

There have likely been a total of 10 species of *Cladopus* plants worldwide, including: *C.austrosinensis*, *C.doianus*, *C.fukienensis*, *C.fallax*, *C.javanicus*, *C.nymanii*, *C.pierrei*, *C.queenslandicus*, *C.taiensis* and *C.yinggelingensis* (Kato 2008; Lin et al. 2016). Molecular phylogenies of the Cladopus section reveal two major clades: one consisting of *C.fallax*, *C.javanicus*, *C.nymanii*, *C.queenslandicus* and *C.taiensis*, and another including the Chinese and Japanese species (Kita and Kato 2001, 2004; Kato and Kita 2003; Kato et al. 2019). Currently, it is generally accepted that five species of *Cladopus* are distributed in China: *C.austrosinensis*, *C.doianus*, *C.fukienensis*, *C.pierrei* and *C.yinggelingensis* (Li et al. 2024).

During a field survey in Yangjiang City, Guangdong Province, China, in February 2024, we encountered a plant in a stream that initially appeared similar to members of the *Cladopus* genus. However, upon more thorough examination, unique features were revealed, suggesting its potential as a previously unknown species. Subsequent comprehensive morphological and molecular systematic analyses confirmed that our collected specimens represent a new species, which we have named *Cladopusyangjiangensis*.

## ﻿Materials and methods

### ﻿Morphological description

The morphological description of the new species was based on the study of specimens collected in a variety of spots in 2024. A stereoscopic zoom microscope (Carl Zeiss, Axio zoom. v.16, Germany), equipped with an attached digital camera (Axiocam), and a digital caliper were used to record the sizes of the morphological characters. Field observations provided habitats and phenology for the new species.

### ﻿DNA extraction, amplification and sequencing

In this study, total DNA was extracted from fresh leaves of the new species using a Quick DNA Isolation Kit (Huayueyang, Beijing). The phylogenetic position of the new species was determined by *matK* and whole plastome sequences. The partial plastid *matK* regions (PQ497705, PQ497706) were amplified via polymerase chain reaction (PCR) using TaKaRa Ex Taq polymerase (TaKaRa, Tokyo, Japan) under the following conditions: 3 min at 94 °C; 35 cycles of 30 s at 94 °C, 30 s at 55 °C, 90 s at 72 °C; and 7 min at 72 °C (Koi et al. 2012). The PCR products were treated with Mag-MK 96 Well PCR Products Purification Kit (Sangon Biotech, Shanghai) to remove the extra primers. Sequencing was conducted using the BigDye Terminator v.3.1 Cycle Sequencing Kit (Applied Biosystems) and the ABI 3130xl Genetic Analyser (Applied Biosystems). The primers used for the DNA amplification and the cycle sequencing are listed in Suppl. material 1: table S1. Other parts of the *matK* sequences were extracted using Geneious v.2021.2.2 from the chloroplast sequences deposited in the GenBank based on the annotated chloroplast genome.

### ﻿Genome sequencing, assembly, annotation and analysis

Purified total DNA of *Cladopusyangjiangensis* was fragmented, genome skimming was performed using next-generation sequencing technologies on the Illumina Novaseq 6000 platform with 150 bp paired-end reads and 400 bp insert size by Berry Genomics Co. Ltd. (Beijing, China), and 10 GB of reads was obtained.

The paired-end reads were filtered and assembled into complete plastome using a GetOrganelle v1.7.5.0 (Jin et al. 2020) with appropriate parameters, with K-merset “21,45,65,85,105”, the word size is 0.6. Following previous studies, our workflow includes five key steps: 1. Mapping reads to seed and assembling seed-mapped reads for parameter estimation; 2. Recruiting more target-associated reads through extending iterations; 3. Conducting de novo assembly; 4. Roughly filtering for target-like contigs; 5. Identifying target contigs and exporting all configurations (Camacho et al. 2009; Bankevich et al. 2012; Langmead and Salzberg 2012; Jin et al. 2020). Graphs of the final assembly were visualized by Bandage (Wick et al. 2015) to assess their completeness. Gene annotation was performed using CPGAVAS2 (Shi et al. 2019) and PGA (Qu et al. 2019). The different annotations of protein coding sequences were confirmed using BLASTx. The tRNAs were checked with tRNAscan-SE v2.0.3. Final chloroplast genome maps were created using OGDRAW.

### ﻿Phylogenetic analysis

The phylogenetic relationship was constructed using Maximum likelihood (ML) analyses with the *matK* sequence. In total, 29 samples (Suppl. material 1: table S2) of *Cladopus* were included in our analysis. Two species of *Paracladopus* were used as outgroups. Each individual sequence was aligned using MAFFT 7.310 (Katoh and Standley 2013) with default settings. All missing data were treated as gaps. The best nucleotide substitution model according to the Bayesian Information Criterion (BIC) was K3Pu+F+R2, which was selected by ModelFinder (Kalyaanamoorthy et al. 2017) implemented in IQTREE v.1.6.8. Maximum likelihood phylogenies were inferred using IQ-TREE (Nguyen et al. 2015) under the model automatically selected by IQ-TREE (‘Auto’ option in IQ-TREE) for 2000 ultrafast (Minh et al. 2013) bootstraps. Bayesian Inference phylogenies were inferred using MrBayes 3.2.6 (Ronquist et al. 2012) under K3Pu+F+R2 model (2 parallel runs, 2000000 generations).

To construct a phylogenetic tree based on plastome sequences, a total of 29 plastome sequences of *Cladopus*, *Terniopsis*, *Polypleurum*, *Paracladopus*, *Marathrum*, *Hydrobryum*, *Cratoxylum*, and *Apinagia* were included in our analysis (Suppl. material 1: table S3). *Cratoxylumcochinchinense* was used as outgroup. Each individual locus was aligned using MAFFT 7.310 (Katoh and Standley 2013) with default settings. The best nucleotide substitution model according to the Bayesian Information Criterion (BIC) was TVM+F+R4, which was selected by ModelFinder (Kalyaanamoorthy et al. 2017) implemented in IQTREE v.1.6.8. Maximum likelihood phylogenies were inferred using IQ-TREE (Nguyen et al. 2015) under the model automatically selected by IQ-TREE (‘Auto’ option in IQ-TREE) for 2000 ultrafast (Minh et al. 2013) bootstraps. Bayesian Inference phylogenies were inferred using MrBayes 3.2.6 (Ronquist et al. 2012) under the GTR+F+I+G4 model (2 parallel runs, 2000000 generations), in which the initial 25% of sampled data were discarded as burn-in. Phylograms were visualized in ChiPlot (Xie et al. 2023).

## ﻿Results

### ﻿Characteristics of *Cladopusyangjiangensis* plastome

**Figure 1.**
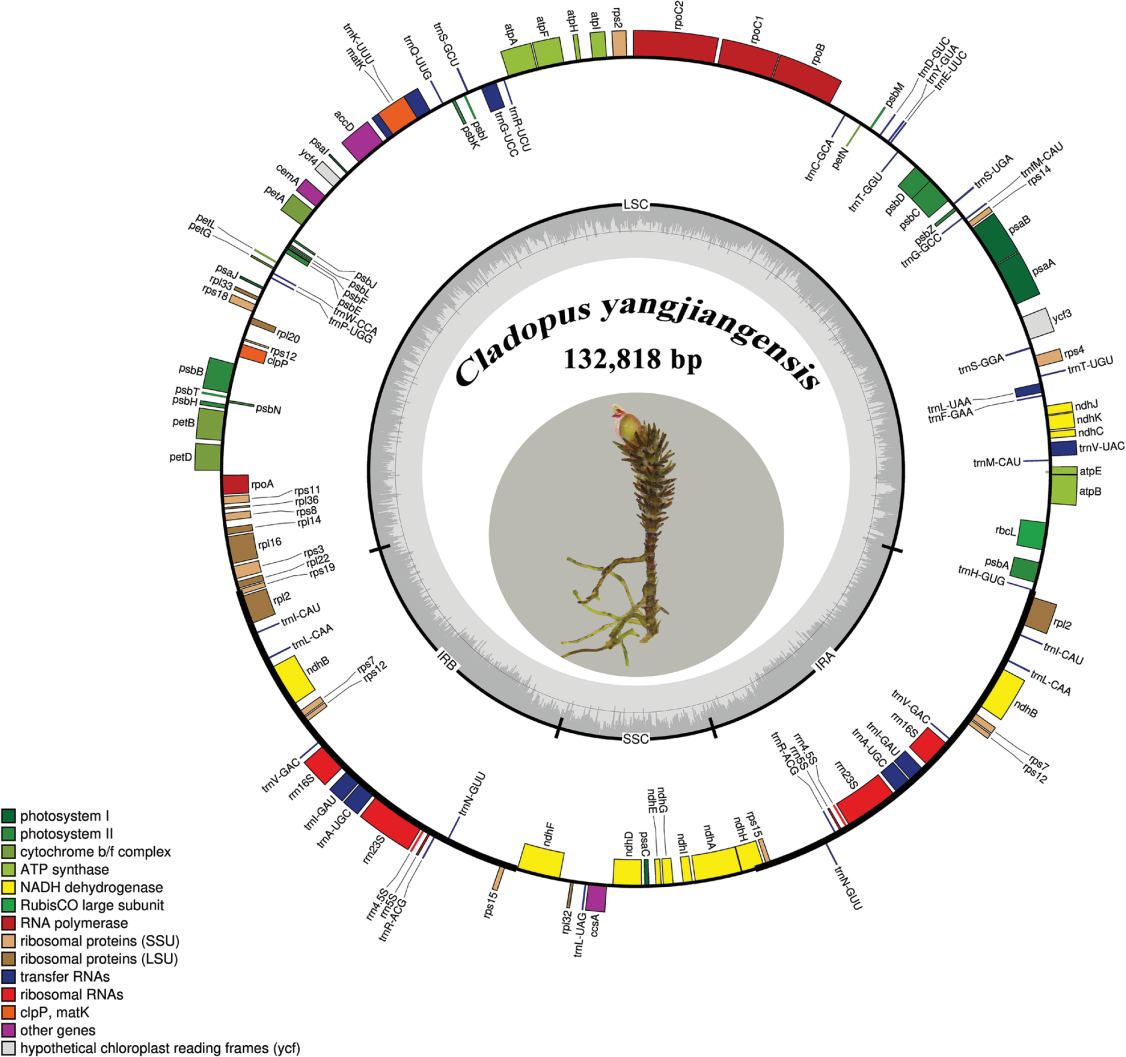
Circular gene map of the plastid genome of *Cladopusyangjiangensis*. Genes inside the circle are transcribed clockwise, while those drawn outside are transcribed counterclockwise. Genes are color-coded according to their functional groups. The circle inside the GC content graph marks the 50% threshold.

The chloroplast genome of *Cladopusyangjiangensis* is 132,818 bp in length (Fig. 1), which exhibits a typical quadripartite structure, comprising a pair of IR regions (20,881 bp) divided by an SSC region (12,343 bp) and an LSC (78,713 bp) region. The overall GC content of the genome was 35.14%, while the GC content of LSC, SSC, and IR regions were 31.99%, 27.93%, and 42.35%, respectively. A total of 108 unique genes were identified in the plastome, it contains 74 protein-coding genes, 30 tRNAs, and 4 rRNAs. A total of 16 genes were duplicated in the IR regions, including *ndhB*, *rpl2*, *rps7*, *rps12*, *rps15*, *rrn4.5S*, *rrn5S*, *rrn16S*, *rrn23S*, *trnA*-*UGC*, *trnI*-*GAU*, *trnI*-*CAU*, *trnL*-*CAA*, *trnN*-*GUU*, *trnR*-*ACG*, *trnV*-*GAC* (Table 1). There were six genes lost, including *rpl23*, *trnT-CGU*, *infA*, *ycf*15, and uncommon losses of *ycf1* and *ycf2*. The annotated plastome was documented in GenBank (PQ510206).

**Table 1.** Gene contents of the plastid genome of *Cladopusyangjiangensis*.

Category, Group of Genes	Gene Names
**Photosynthesis**:	
Subunits of photosystem I	*psaA*, *psaB*, *psaC*, *psaI*, *psaJ*
Subunits of photosystem II	*psbA*, *psbB*, *psbC*, *psbD*, *psbE*, *psbF*, *psbH*, *psbI*, *psbJ*, *psbK*, *psbL*, *psbM*, *psbN*, *psbT*, *psbZ*
Subunits of NADH dehydrogenase	ndhA*, ndhB*(2), *ndhC*, *ndhD*, *ndhE*, *ndhF*, *ndhG*, *ndhH*, *ndhI*, *ndhJ*, *ndhK*
Subunits of cytochrome b/f complex	*petA*, petB*, petD*, *petG*, *petL*, *petN*
Subunits of ATP synthase	*atpA*, *atpB*, *atpE*, atpF*, *atpH*, *atpI*
Large subunit of rubisco	*rbcL*
**Self-replication**:	
Subunits of RNA polymerase	*rpoA*, *rpoB*, rpoC1*, *rpoC2*
Proteins of large ribosomal subunit	*rpl14*, rpl16*, rpl2*(2), *rpl20*, *rpl22*, *rpl32*, *rpl33*, *rpl36*
Proteins of small ribosomal subunit	*rps11*, rps12*(2), *rps14*, *rps15* (2), *rps18*, *rps19*, *rps2*, *rps3*, *rps4*, *rps7* (2), *rps8*
Transfer RNAs	trnA-UGC*(2), *trnC-GCA*, *trnD-GUC*, *trnE-UUC*, *trnF-GAA*, *trnG-GCC*, trnG-UCC*, *trnH-GUG*, *trnI-CAU* (2), trnI-GAU*(2), trnK-UUU*, *trnL-CAA* (2), trnL-UAA*, *trnL-UAG*, *trnM-CAU*, *trnN-GUU* (2), *trnP-UGG*, *trnQ-UUG*, *trnR-ACG* (2), *trnR-UCU*, *trnS-GCU*, *trnS-GGA*, *trnS-UGA*, *trnT-GGU*, *trnT-UGU*, *trnV-GAC* (2), trnV-UAC*, *trnW-CCA*, *trnY-GUA*, *trnfM-CAU*
Ribosomal RNAs	*rrn16S* (2), *rrn23S* (2), *rrn4.5S* (2), *rrn5S* (*2*)
**Other genes**:	
Maturase	*matK*
Protease	*clpP*
Envelope membrane protein	*cemA*
c-type cytochrome synthesis gene	*ccsA*
Acetyl-CoA carboxylase	*accD*
**Unknown function**:	
Conserved open reading frames	ycf3*, *ycf4*

Notes: *gene with one introns; Gene (2): Number of copies of multi-copy genes.

### ﻿Comparative analysis of the plastomes

A comparative analysis was conducted on the plastid genomes of all six known species within the genus *Cladopus* found in China (Table 2). The plastid genome sizes varied slightly, ranging from 132,046 bp in *C.austrosinensis* to 132,907 bp in *C.yinggelingensis*, with an overall average size of 132,739 bp. Each plastid genome displayed the typical quadripartite structure, consisting of a large single-copy (LSC) region, a small single-copy (SSC) region, and two inverted repeat (IR) regions. The LSC region was the largest, comprising approximately 59.1% to 59.4% of the total plastid genome length, followed by the IR regions at 31.4% to 31.6%, and the SSC region at 9.2% to 9.3%. This consistent structural organization across species underscores the conserved nature of the plastid genome in *Cladopus*.

**Table 2.** Basic features of the plastid genomes of all known species within the genus *Cladopus* in China.

Species	Accession no.	Number of Genes			Length (bp)				GC content (%)
		PCGs	tRNA	rRNA	Total	LSC	SSC	IR	
*C.austrosinensis*	PQ510207	73	29	4	132,046	77,993 (~59.1%)	12,295 (~9.3%)	20,879 × 2 (~31.6%)	34.98
*C.doianus*	PQ510208	75	30	4	132,896	78,967 (~59.4%)	12,211 (~9.2%)	20,859 × 2 (~31.4%)	34.88
*C.fukienensis*	NC_082923.1	74	30	4	132,834	78,741 (~59.3%)	12,331 (~9.3%)	20,881 × 2 (~31.4%)	35.39
*C.pierrei*	NC_082924.1	74	30	4	132,893	78,865 (~59.3%)	12304 (~9.3%)	20,862 × 2 (~31.4%)	34.92
*C.yinggelingensis*	NC_082925.1	74	30	4	132,907	78,878 (~59.3%)	12,311 (~9.3%)	20,859 × 2 (~31.4%)	35.36
*C.yangjiangensis*	PQ510206	74	30	4	132,818	78,713 (~59.3%)	12,343 (~9.3%)	20,881 × 2 (~31.4%)	35.14

The GC content was consistent across the species, ranging from 34.88% to 35.39%, with a mean value of 35.12%. This homogeneity in GC content suggests a stable evolutionary trajectory with limited genomic rearrangements or substitutions. The number of protein-coding genes varied slightly, ranging from 73 to 75. The number of transfer RNA genes was either 29 or 30, while the number of ribosomal RNA genes remained consistently four in each species. These gene counts reflect the core functionality of the plastid genome, which is primarily involved in photosynthesis and genetic expression.

### ﻿Phylogenetic analysis

**Figure 2.**
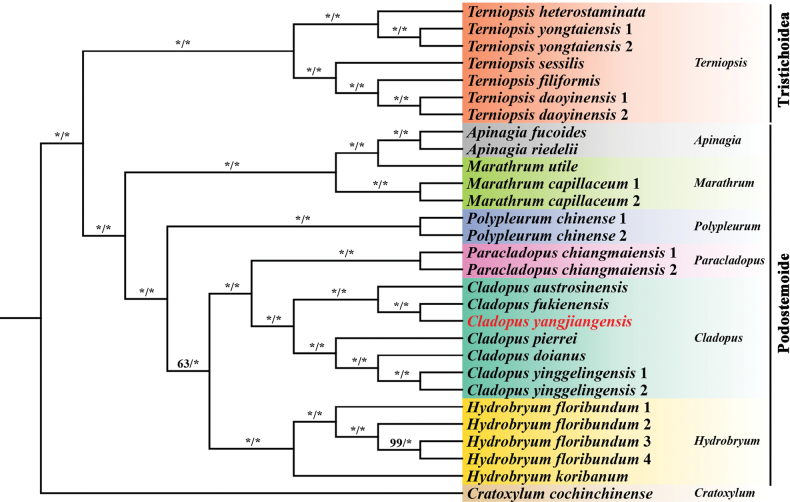
Phylogenetic consensus tree of 29 complete plastid sequences derived from the Podostemaceae species in the genera *Cladopus*, *Terniopsis*, *Polypleurum*, *Paracladopus*, *Marathrum*, *Hydrobryum*, and *Apinagia*. Numbers above and below branches indicate RAxML (left) bootstrap probabilities (BP) and Bayesian (right) posterior probabilities (PP), respectively. Cratoxylumcochinchinense was included as an outgroup. * indicates bootstrap probabilities (BP) = 100 and Bayesian posterior probabilities (PP) = 1.00, unless otherwise indicated at nodes.

**Figure 3.**
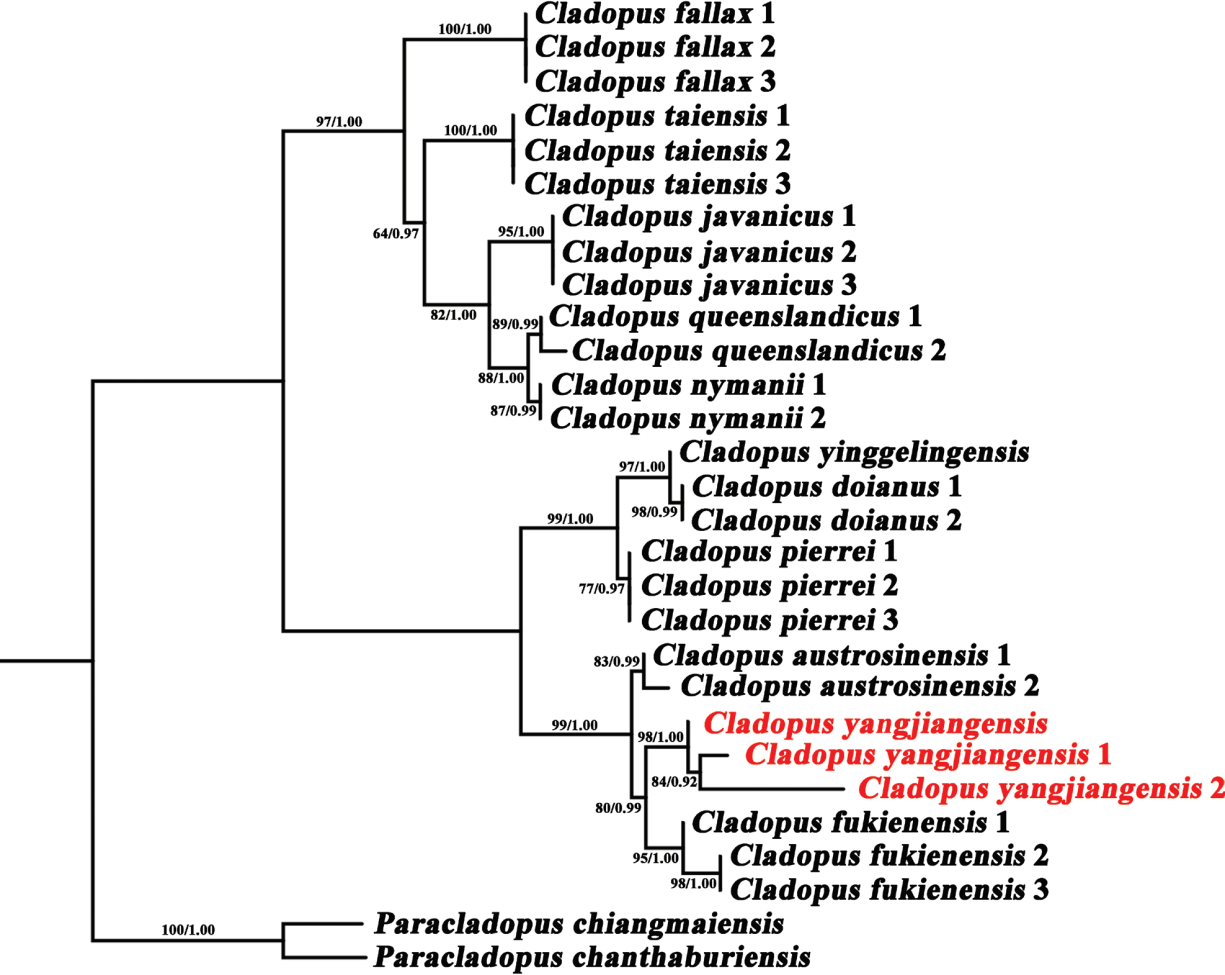
Phylogenetic tree of 29 sequences based on *matK* genes. The left tree is Maximum likelihood tree and the number above branches indicate RAxML bootstrap probabilities (BP). The right tree is MrBayes tree and the number above branches indicate Bayesian posterior probabilities (PP), respectively. *Paracladopus* were outgroups of the trees.

The present study confirms *Cladopusyangjiangensis* as a new species based on phylogenetic analysis, using Maximum likelihood (ML) and Bayesian Inference (BI) methods on plastome data and the *matK* sequence. The phylogenetic tree based on plastome data includes seven genera from the subfamily Podostemoideae and *Terniopsis* from the subfamily Tristichoideae (Fig. 2), thereby explicitly illustrating the relationships among these taxa. *Cladopus* is shown to be a monophyletic group within Podostemoideae, comprising two subclades. The new species is clearly distinctly separated from the most closely related species, *C.fukienensis*, with strong support (BP = 100, PP = 1.00), and is positioned within a clade that also contains *C.austrosinensis*. The same pattern was found in the *matK* tree (Fig. 3), constructed from 29 samples of the of the *Cladopus* species, providing a comprehensive view of the phylogenetic relationship within the genus.

### ﻿Taxonomic treatment

#### ﻿ Cladopus yangjiangensis

Taxon classificationPlantaeMalpighialesPodostemaceae

X.T.Liu, G.Di Chen & B.Hua Chen sp. nov.

B9475B79-D5E7-513D-9E3A-C635165C4599

urn:lsid:ipni.org:names:77352702-1

**Figure 4.**
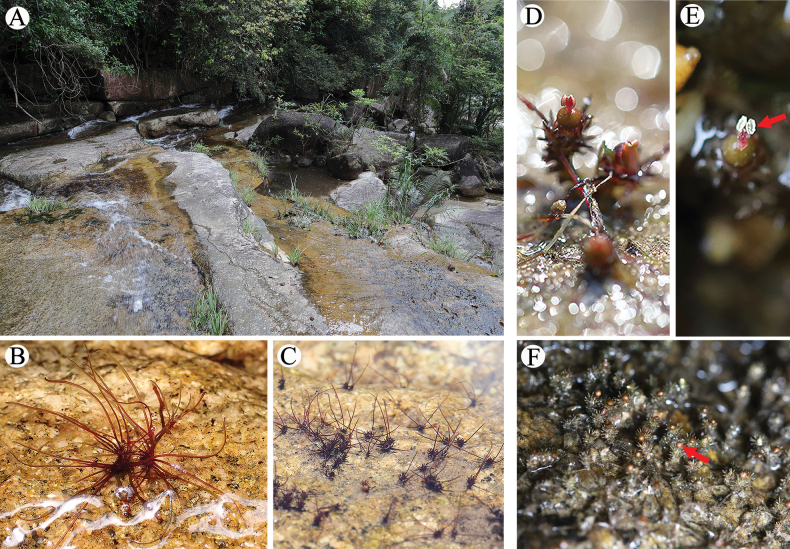
*Cladopusyangjiangensis***A** habitat (Photographed by Guo-Di Chen) **B** root with tufts of leaves, leaves linear, brick red in color **C** plants in bud adhering to rock surface **D** flowering shoot **E** top view of flower (red arrow pointing to the stamen) **F** mature fruits.

**Figure 5.**
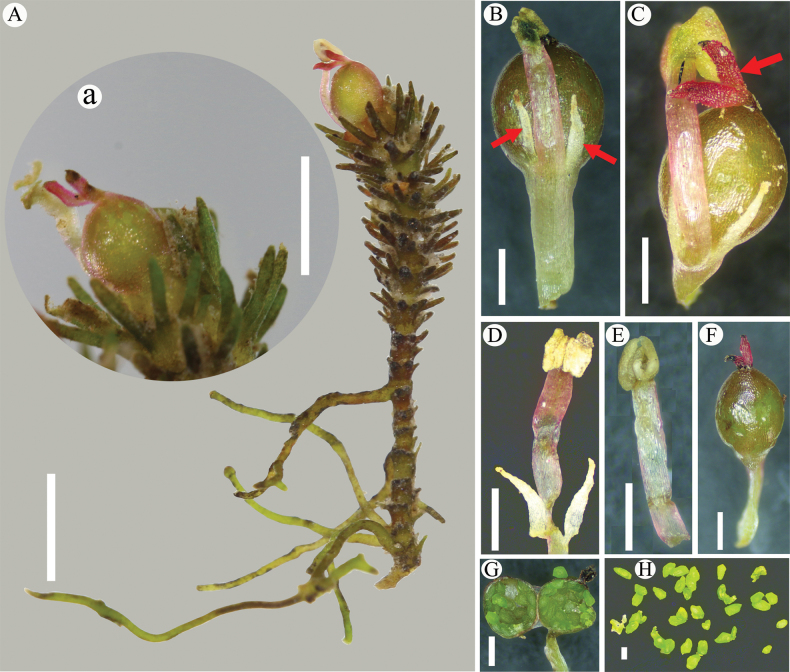
*Cladopusyangjiangensis***A** flowering shoot on roots, inverted tower shape (reproductive leaves (a.k.a. bracts) imbricate, finger-like) a flower with 2 stigmas, a stamen (stamen clearly longer than ovary) **B** stamens, arrow points to two tepals, fused to ovary **C** gynoecia, arrow points to reddish, ribbon-shaped stigmas **D** stamen and tepals (tepals attached to filament bases on either side) **E** stamen **F** gynoecium with ellipsoid ovary, no bracts **G** longitudinal ovary section **H** ovules. Scale bars: 2 mm (**A**); 500 μm (**A, B, C, D, E, F, G**); 200 μm (**H**).

**Figure 6.**
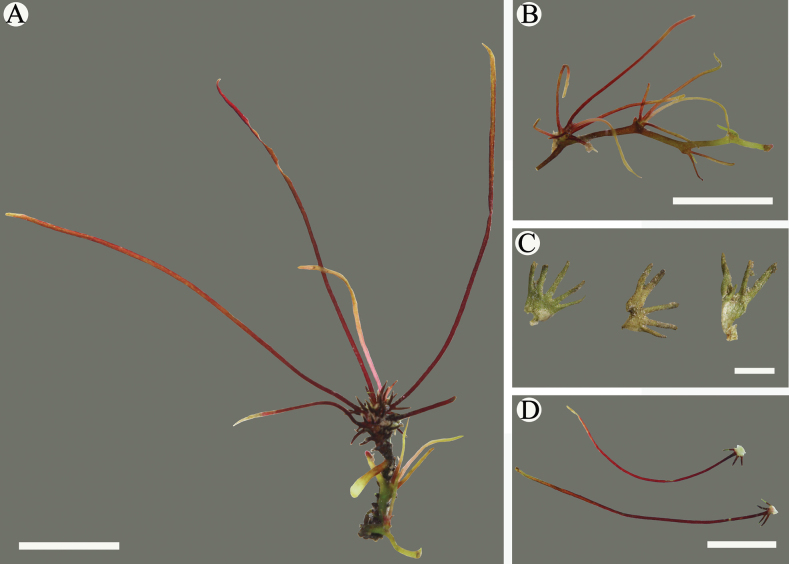
*Cladopusyangjiangensis***A** vegetative shoot **B** tufts of leaves on root (clustered, roots subterete, linear leaves) **C** abaxial and adaxial views of the upper leaves of reproductive shoots **D** leaves with some lobes markedly long at the base of vegetative shoots. Scale bars: 5 mm (**A, B**); 1 mm (**C, D**).

**Figure 7.**
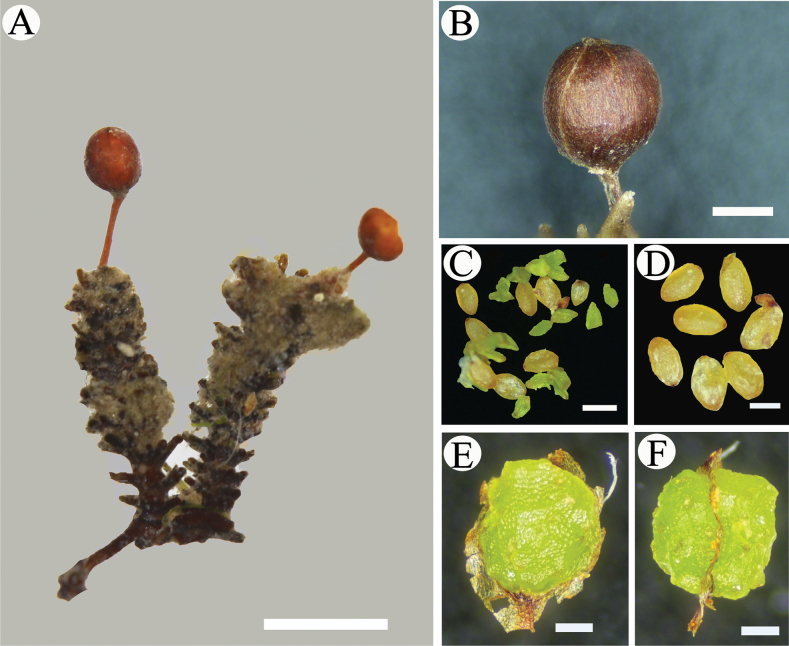
*Cladopusyangjiangensis***A** fruiting shoots on root **B** stalked capsule, tan, globose, smooth **C** fertile seeds and ovules in a mature fruit **D** fertile seeds **E, F** adaxial and lateral views of placenta. Scale bars: 2 mm (**A**); 400 μm (**B**); 200 μm (**C, D, E, F**).

**Figure 8.**
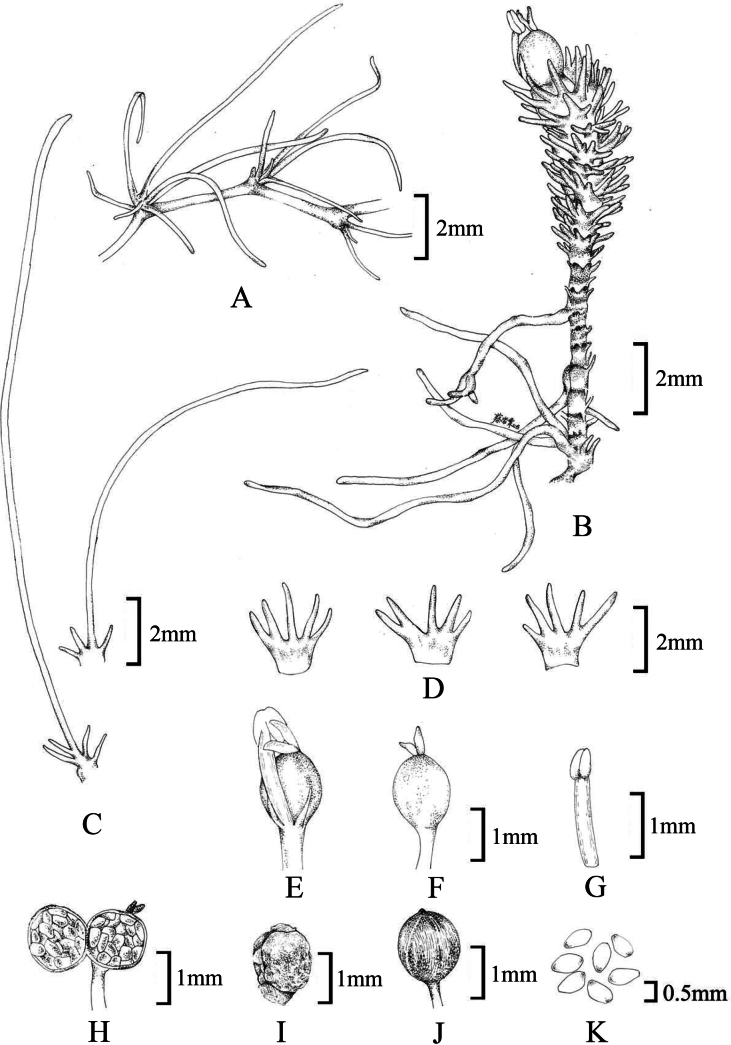
Illustration of *Cladopusyangjiangensis***A** tufts of leaves on root **B** flowering shoot on roots **C** leaves with some lobes markedly long at the base of vegetative shoots **D** abaxial and adaxial views of leaves on the upper part **E** flower with 2 stigmas, a stamen and 2 tepals **F** gynoecium without bracts, ovary ellipsoid **G** stamen **H** longitudinal section of the ovary **I** adaxial view of the placenta **J** stalked capsule, globose, smooth **K** seeds.

Figs 4 , 5 , 6 , 7 , 8

##### Diagnosis.

*Cladopusyangjiangensis* shares certain similarities with *C.fukienensis* and *C.austrosinensis*, such as comparable ovary lengths, and analogous stigma and capsule shapes. However, it is distinguished by several unique traits. The leaves of *C.yangjiangensis* are markedly elongated, measuring 18.7–26.7 mm, which is substantially longer than those of *C.fukienensis* (1.3–5.0 mm) and *C.austrosinensis* (up to 6 mm). The flowering shoots of *C.yangjiangensis* also exceed those of other two species, ranging from 4.2 to 13.1 mm, compared to 3.5–6.0 mm in *C.fukienensis* and 1.6–3.5 mm in *C.austrosinensis*. Additionally, *C.yangjiangensis* produces a greater number of bracts, with counts ranging from 20 to 54, in contrast to 12–36 in *C.fukienensis* and 8–14 in *C.austrosinensis*. Finally, the root width of *C.yangjiangensis* is notably narrower, at about 0.4 mm, compared to 0.4–1.3 mm in *C.fukienensis* and 0.5–1.3 mm in *C.austrosinensis* (Table 3).

**Table 3.** Comparison of three congeneric species of *Cladopus*.

Characteristics	*C.austrosinensis*	*C.fukienensis*	*C.yangjiangensis*
Root width (mm)	0.5–1.0(–1.3)	0.4–1.3	ca. 0.4
The number of leaves	to 5	2–5	3–8
Leaves length (mm)	to 6.0	1.3–5.0	18.7–26.7
Flowering shoots length (mm)	1.6–3.0(–3.5)	3.5–6.0	4.2–13.1
The number of bracts	8–12(–14)	12–19(–36)	20–54
Finger-like lobes	3–9	3–7	3–5
The size of spathella (mm)	/	1.3–1.9 × 0.9–1.4	1.7 × 0.3
The morphology and length of tepals (mm)	linear, 1.0–1.5	linear or subulate, 0.6–0.7	broadly linear, acuminate at end, 0.7–1.1
Stamen length (mm)	ca. 1.5	ca. 1.3	1.2–2.4
Ovary length (mm)	1.0–1.5	1.1–1.5	1.2–1.7
Stigma length (mm)	ca. 0.6	0.4–0.5	0.3–0.4
Ovules locule	25–34	25–35	30–45
Length of stamens vs. length of ovaries	Equal	shorter or equal	longer
Capsule stalk length (mm)	1.2–1.7	(0.5–)1.2–2.8	1.2–1.3(–1.7)
The size of capsule (mm)	ca. 1.5	1.0–1.3 × 0.8–1.3	1.1–1.4 × 0.9–1.2
The size of seed (mm)	0.2–0.3 × 0.1–0.2	0.3–0.5 × 0.2–0.3	0.3–0.5 × 0.1–0.3
Distribution	South China	South & southeast China	South China

##### Type.

China • Guangdong: Yangjiang City Yangxi County, Tangkou Town, Tongyou village, elevation 200 m, 21°49'N, 111°28'E, 24 December 2023, *XT Liu & GD Chen 0001* (***Holotype*** FNU! barcode FNU0041314; isotype FNU! barcode FNU0041315).

##### Description.

Perennial aquatic herb; roots narrowly ribbon-like, compressed, dorsiventral, succulent, ca. 0.4 mm width, 0.1–0.2 mm thick, adhere to the surface of underwater rocks, brick-red during winter, dark green during reproduction; stem short, arising from root branch axils (Fig. 4); flowering shoots obpyramidal, solitary, erect, 4.2–13.1 mm tall; leaves linear, in rosette on vegetative shoots, 3–8 in number, 18.7–26.7 mm long, deciduous at flowering; leaves on reproductive shoots palmate (also known as bracts), lobes 3–5 digitate, central lobe long, columns two, opposite, overlapping, bracts 20–54, upper leaves larger, diminishing towards apex, 1.5–1.9 × 1.1–2.0 mm, lobes rigid and coarse after water loss; flowers bisexual, solitary at fertile branch apex, enclosed in pale red spathe during early development; spathella globose, acumen short, 1.7 × 0.3 mm; tepals two, broadly linear, acuminate, 0.7–1.1 mm long; stamen single, 1.2–2.4 mm long; filament nearly cylindrical, slightly flattened, 0.9–1.3 mm long; anthers two, elliptical, yellow, ~ 0.6–0.9 mm long; Ovary single, pale yellow-green, ellipsoid, two-chambered, 1.2–1.7 × 0.9–1.1 mm; ovules ovate, 15–23 per chamber, attached to entire placenta (Figs 5, 6); capsule brownish, globose, smooth, 1.1–1.4 mm long; fruit stalk 1.2–1.7 mm long; seeds small, yellow, narrowly ovoid, 0.3–0.5 × 0.1–0.3 mm (Fig. 7).

##### Distribution and habitat.

Many other plants grow in the surrounding habitat, whose tree layer includes *Archidendronclypearia* (Fabaceae), *Engelhardiaroxburghiana* (Juglandaceae), *Aporosadioica* (Phyllanthaceae), *Zanthoxylumavicennae* (Rutaceae), *Sterculialanceolata* (Malvaceae) and others; the shrub layer includes *Acronychiapedunculata* (Rutaceae), *Rhaphiolepisindica* (Rosaceae), *Rubusleucanthus* (Rosaceae, *Ficuspyriformis* (Moraceae), *Glochidionlanceolarium* (Phyllanthaceae), *Garciniaoblongifolia* (Clusiaceae), *Melastomasanguineum* (Melastomataceae), *Saurauiatristyla* (Actinidiaceae), *Adina pilulifera* (Rubiaceae), *Pavettahongkongensis* (Rubiaceae), and others; the vegetation layer includes *Blechnopsisorientalis* (Blechnaceae), *Plenasiumvachellii* (Osmundaceae), *Acorusgramineus* (Acoraceae), *Pandanusaustrosinensis* (Pandanaceae), *Alpiniahainanensis* (Zingiberaceae), *Thysanolaenalatifolia* (Poaceae), *Miscanthusfloridulus* (Poaceae), *Pentasachmecaudata* (Apocynaceae) and others; and some exotic plants include *Stauntoniaobovatifoliola* (Lardizabalaceae), *Phaneraerythropoda* (Fabaceae), *Roureamicrophylla* (Connaraceae) and others.

##### Phynology.

Flowering and fruiting season in November to February of the following year.

##### Etymology.

The Yang Jiang Chuan Tai Cao (阳江川苔草).The epithet *yangjiangensis* (阳江) refers to Yangjiang City, Guangdong Province, South China, where this new species was found.

##### Conservation status.

According to our investigation, *Cladopusyangjiangensis* was only found in a stream in Yangjiang City, Guangdong Province, China, and hence we suggest its placement in the Data Deficient category of IUCN (2022). In addition, according to the Updated List of National Key Protected Wild Plants (Decree No. 15) by the country’s State Forestry and Grassland Administration and the Ministry of Agriculture and Rural Affairs, all of the known genera of Podostemaceae found in China are classified as under national secondary protection. This new species should also be included on the national secondary protection list during the upcoming revision process.

## ﻿Discussion

The discovery of *Cladopusyangjiangensis* enriches our understanding of the diversity of the Podostemaceae in East Asia, showcasing unique morphological and genetic characteristics that distinguish it from other species in the genus. Currently, five species of *Cladopus* are recognized in China: *C.austrosinensis*, *C.doianus*, *C.fukienensis*, *C.pierrei*, and *C.yinggelingensis*. Among these, *C.austrosinensis* is found in Hainan and Guangdong, while *C.fukienensis* has a wider distribution, occurring in Fujian, Guangdong, Hong Kong, and other regions.

Previous phylogenetic analyses demonstrated that sequences identified as *Cladopusaustrosinensis* (accession numbers AB104560.1, AB104559.1, and LC 144911.1) are paraphyletic and interspersed with sequences from *C.fukienensis*, hinting at unrecognized species diversity within this group (Kita and Kato 2004; Kato et al. 2017). Our recent *matK* gene sequencing of specimens collected from Boluo County, near the original discovery site in Huizhou, east-central Guangdong, confirmed their identity as genuine *C.austrosinensis*. Subsequent fieldwork in Yangjiang City, southern Guangdong, revealed a *Cladopus* species with distinct morphological characteristics, which are different from *C.austrosinensis*. Genetic analyses align this population with the southern Guangdong samples (accession no. AB104559.1), forming a monophyletic group. This supports the identification of the Yangjiang population as a distinct species, previously misidentified as *C.austrosinensis*. We propose the designation of this population as a new species, *Cladopusyangjiangensis*, thereby resolving the phylogenetic discrepancies highlighted in earlier studies (Kita and Kato 2004; Kato et al. 2017).

*Cladopusyangjiangensis* exhibits several unique morphological features that distinguish it within the East Asian *Cladopus* species. Notably, it possesses exceptionally long flowering shoots, reaching up to 13.1 mm, the longest recorded among *Cladopus* species in East Asia, although elsewhere species such as *C.javanicus* and *C.queenslandicus* have longer flowering shoots that can reach lengths of 30–70 mm and 30–90 mm, respectively (Kato et al. 2019). It also has extremely long leaves in the vegetative state (18.7–26.7 mm), the longest among all *Cladopus* species in East Asia (Lin et al. 2016; Kato et al. 2017; Werukamkul et al. 2018). Other distinctive features include its inverted tower-shaped flowering shoots, distinct bracts, roots, stamens, and stigmas, as confirmed through detailed microscopic anatomical observations. Furthermore, the roots of *C.yangjiangensis*, like other Podostemaceae, deviate from the typical radially symmetric cylindrical roots of most angiosperms. They are dorsiventral and range from compressed subcylindrical to ribbon-like forms (Cusset 1992; Kato and Hambali 2001; Rutishauser and Pfeifer 2002). Specifically, *C.yangjiangensis* is notable for having thinner roots, adding to its distinctive profile within the genus.

Genetic analysis further supports the distinctiveness of *C.yangjiangensis*. The chloroplast genomes of *Cladopus* species, including *C.yangjiangensis*, are characterized by the extensive loss of the *ycf1* and *ycf2* genes, a phenomenon observed across the Podostemaceae family. These genes, among the longest in the chloroplast genome (Ren et al. 2020), are typically about 20 kb in length (Dong et al. 2015; De Santana Lopes et al. 2018), and their loss may contribute to the reduced chloroplast genome size in this family. While gene loss in chloroplast genomes is common, the absence of *ycf1* and *ycf2* genes, which encoded essential cellular functions, suggests potential gene transfer to nuclear or mitochondrial genomes (Cauz-Santos et al. 2017). However, homologs have yet to be identified in the nuclear genome (Drescher et al. 2000). Previous studies have indicated that plants in moist, shady environments often lack the *ycf1* gene (Wen et al. 2022), suggesting a similar environmental influence on Podostemaceae. Interestingly, *ycf1* gene loss is more commonly associated with parasitic and heterotrophic plants (Li et al. 2020; Yudina et al. 2021), making its occurrence in autotrophic *Cladopus* species noteworthy. Additional chloroplast genome data from similar habitats are needed to validate the correlation between *ycf* gene presence and environmental conditions.

The discovery of *C.yangjiangensis* and recent findings of new and newly recorded Podostemaceae species in China, such as *Polypleurumchinense* (Chen et al. 2022), *Paracladopuschiangmaiensis* (Wu et al. 2022), and *Terniopsisyongtaiensis* (Zhang et al. 2022), suggest that the wild resources of Podostemaceae in China have been significantly underestimated. It is anticipated that further in-depth investigations will uncover additional distribution sites of Podostemaceae within China, enhancing our understanding of this unique and diverse family.

### ﻿Key to the species of *Cladopus*

**Table:** 

1	Flowering shoots 25.0–90.0 mm long	**2**
–	Flowering shoots 1.0–13.1 mm long	**3**
2	Bracts digitate; roots 2.0–4.0 mm wide; ca. 100 ovules per ovary	***Cladopusjavanicus***
–	Bract trilobed; roots 0.5–2.0 mm wide; ca. 50–80 ovules per ovary	***Cladopusqueenslandicus***
3	Roots > 2.0 mm wide	**4**
–	Roots 0.2–2.0 mm wide	**8**
4	Bracts 15–20	***Cladopusyinggelingensis***
–	Bracts 4–12	**5**
5	Stamens distinctly longer than ovary	***Cladopusnymanii***
–	Stamens as long as or slightly longer than ovary	**6**
6	Stigmas narrowly fan-shaped with dilate apex, obovate-spatulate	***Cladopusdoianus***
–	Stigmas linear	**7**
7	Flowering shoots ca. 6.0 mm long; bracts 8–10	***Cladopuspierrei***
–	Flowering shoots 1.0–2.0 mm long; bracts 4–6	***Cladopustaiensis***
8	Stamens 0.3–0.6 mm long	***Cladopusfallax***
–	Stamens 1.5–2.4 mm long	**9**
9	Flowering shoots 1.6–3.0(–3.5) mm long; bracts 8–12	***Cladopusaustrosinensis***
–	Flowering shoots 3.5–13.1 mm long; bracts 12–54	**10**
10	Leaves 1.3–5.0 mm long; stamens as long as or slightly shorter than ovary	***Cladopusfukienensis***
–	Leaves 18.7–26.7 mm long; stamens distinctly longer than ovary	***Cladopusyangjiangensis***

## Supplementary Material

XML Treatment for Cladopus yangjiangensis

## ﻿Additional information

### Author ORCIDs

Bing-Hua Chen https://orcid.org/0000-0001-5422-7532

### Data availability

All of the data that support the findings of this study are available in the main text or Supplementary Information.

## Supplementary materials

Supplementary material 1 Supplementary data Data type doc Explanation note **table S1.** Primers used in this study. **table S2.** The information of phylogenetic analysis for the species used in the present study (*matK*). **table S3.** The information of phylogenetic analysis for the species used in the present study (plastid genome). https://binary.pensoft.net/file/1188257This dataset is made available under the Open Database License (http://opendatacommons.org/licenses/odbl/1.0/). The Open Database License (ODbL) is a license agreement intended to allow users to freely share, modify, and use this Dataset while maintaining this same freedom for others, provided that the original source and author(s) are credited.Miao Zhang, Xiu-Ting Liu, Min Tian, Zhang-Xue Chen, Ying-Lin Huang, Guo-Di Chen, Bing-Hua Chen
