# Supplementary material for: ﻿Cladopusyangjiangensis (Podostemaceae), a new species from Guangdong, South China, redefining the phylogenetic relationships within Cladopus
Source: PhytoKeys. 2024 Dec 2;249:231–49. doi: 10.3897/phytokeys.249.140342 (PMC11629083; doi:10.3897/phytokeys.249.140342)
Supplement: Supplementary material 1 — Supplementary data [file phytokeys-249-231_article-140342__-s001.doc]

**Appendix A. Supplementary data**

Table S1. Primers used in this study

| Name | Sequence (5′–3′) | Direction | Source |
| --- | --- | --- | --- |
| PodMK-F(p.w.d) | TATCGCACTAYGTATCAKTT | F | Koi et al. 2012 |
| PodMK-R | TATCGCACACGGCTTTC | R | Koi et al. 2012 |

Table S2. The information of phylogenetic analysis for the species used in the present study (*matK*)

| Family | Genus | Species | GenBank accession number |
| --- | --- | --- | --- |
| Podostemaceae | *Cladopus* | *Cladopus fallax 1* | LC380597.1 |
|  |  | *Cladopus fallax* 2 | LC380598.1 |
|  |  | *Cladopus fallax* 3 | LC380596.1 |
|  |  | *Cladopus javanicus* 1 | LC380600.1 |
|  |  | *Cladopus javanicus* 2 | AB066175.1 |
|  |  | *Cladopus javanicu*s 3 | LC380599.1 |
|  |  | *Cladopus nymanii* 1 | AB104577.1 |
|  |  | *Cladopus nymanii* 2 | AB698211.1 |
|  |  | *Cladopus queenslandicus* 1 | AB300702.1 |
|  |  | *Cladopus queenslandicus 2* | AB038199.1 |
|  |  | *Cladopus taiensis* 1 | LC151308.1 |
|  |  | *Cladopus taiensis* 2 | LC151309.1 |
|  |  | *Cladopus taiensis* 3 | LC151307.1 |
|  |  | *Cladopus austrosinensis* 1 | LC144911.1 |
|  |  | *Cladopus austrosinensis* 2 | PQ510207 |
|  |  | *Cladopus fukienensis* 1 | AB179653.1 |
|  |  | *Cladopus fukienensis* 2 | LC144913.1 |
|  |  | *Cladopus fukienensis* 3 | LC144912.1 |
|  |  | *Cladopus pierrei* 1 | LC151302.1 |
|  |  | *Cladopus pierrei* 2 | LC151305.1 |
|  |  | *Cladopus pierrei* 3 | LC151304.1 |
|  |  | *Cladopus yinggelingensis* | NC_082925.1 |
|  |  | *Cladopus doianus* 1 | AB179656.1 |
|  |  | *Cladopus doianus* 2 | AB698209.1 |
|  |  | *Cladopus yangjiangensis* | PQ510206 |
|  |  | *Cladopus yangjiangensis* 1 | PQ497705 |
|  |  | *Cladopus yangjiangensis* 2 | PQ497706 |
|  | *Paracladopus* | *Paracladopus chiangmaiensis* | AB293560.1 |
|  |  | *Paracladopus chanthaburiensis* | AB300701.1 |

Table S3. The information of phylogenetic analysis for the species used in the present study ( plastid genome)

| Family | Genus | Species | GenBank accession number |
| --- | --- | --- | --- |
| Podostemaceae | *Cladopus* | *Cladopus austrosinensis* | PQ510207 |
|  |  | *Cladopus doianus* | PQ510208 |
|  |  | *Cladopus yangjiangensis* | PQ510206 |
|  |  | *Cladopus yinggelingensis* 1 | NC_082925.1 |
|  |  | *Cladopus yinggelingensis* 2 | OR393189.1 |
|  |  | *Cladopus pierrei* | NC_082924.1 |
|  |  | *Cladopus fukienensis* | NC_082923.1 |
|  | *Polypleurum* | *Polypleurum chinense* 1 | NC_070353.1 |
|  |  | *Polypleurum chinense* 2 | OL944404.1 |
|  | *Paracladopus* | *Paracladopus chiangmaiensis* 1 | MZ645928.1 |
|  |  | *Paracladopus chiangmaiensis* 2 | NC_061663.1 |
|  | *Marathrum* | *Marathrum utile* | MN165814.1 |
|  |  | *Marathrum capillaceum* 1 | MN165813.1 |
|  |  | *Marathrum capillaceum* 2 | NC_060291.1 |
|  | *Hydrobryum* | *Hydrobryum koribanum* | PP882865 |
|  |  | *Hydrobryum floribundum* 1 | OR393191.1 |
|  |  | *Hydrobryum floribundum* 2 | PP882864 |
|  |  | *Hydrobryum floribundum* 3 | NC_082926.1 |
|  |  | *Hydrobryum floribundum* 4 | OR393190.1 |
|  | *Apinagia* | *Apinagia riedelii* | NC_059684.1 |
|  |  | *Apinagia fucoides* | MN165812.1 |
|  | *Terniopsis* | *Terniopsis yongtaiensis* 1 | OM717943.1 |
|  |  | *Terniopsis yongtaiensis* 2 | NC_066797.1 |
|  |  | *Terniopsis sessilis* | NC_082922.1 |
|  |  | *Terniopsis heterostaminata* | NC_082929.1 |
|  |  | *Terniopsis filiformis* | NC_082928.1 |
|  |  | *Terniopsis daoyinensis* 1 | NC_082927.1 |
|  |  | *Terniopsis daoyinensis* 2 | OR393192.1 |
| Hypericaceae | *Cratoxylum* | *Cratoxylum cochinchinense* | MN399961.1 |
